# Supplementary material for: Eupatin, a Flavonoid, Inhibits Coronavirus 3CL Protease and Replication
Source: Int J Mol Sci. 2023 May 24;24(11):9211. doi: 10.3390/ijms24119211 (PMC10252338; doi:10.3390/ijms24119211)
Supplement: Supplementary file 1 [file ijms-24-09211-s001.zip › ijms-2415737-supplementary.pdf]

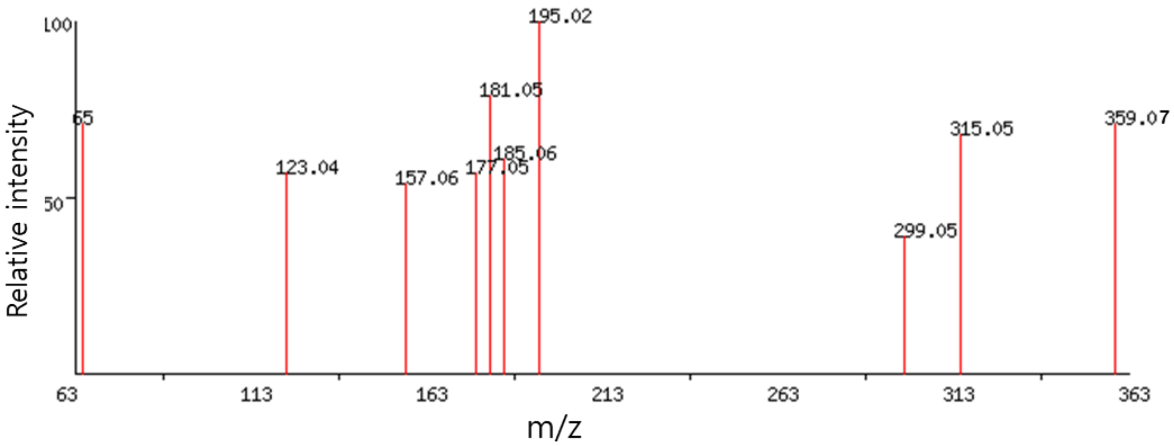

Figure S1. ESI-MS of eupatin

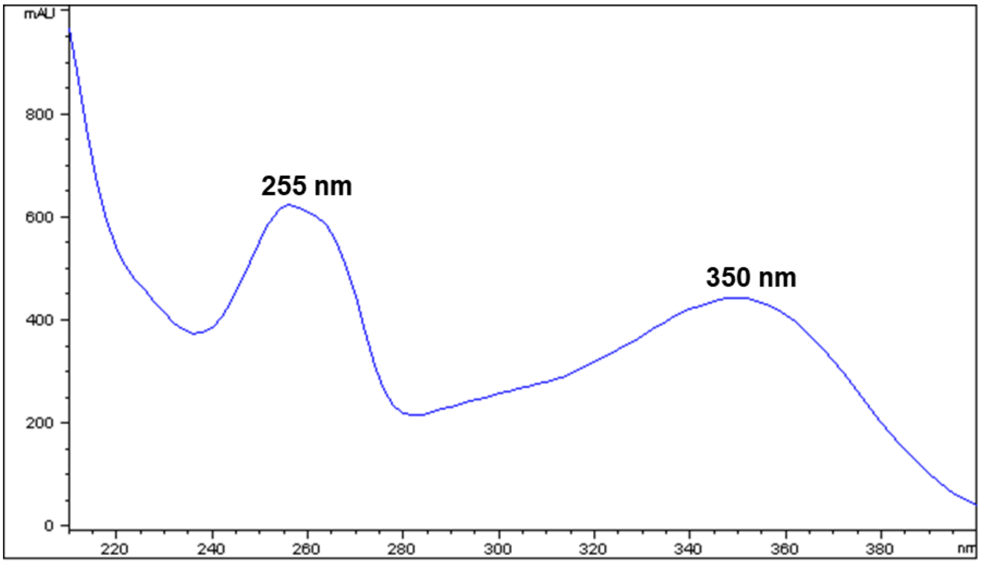

Figure S2. UV spectrum of eupatin

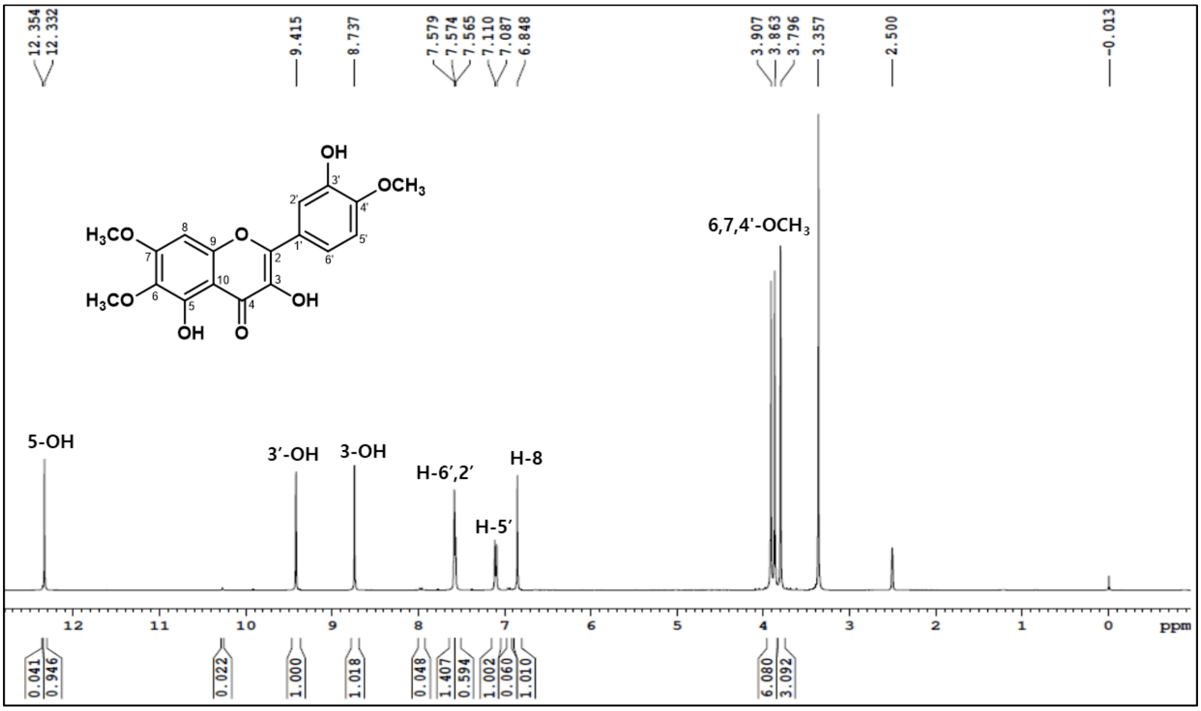

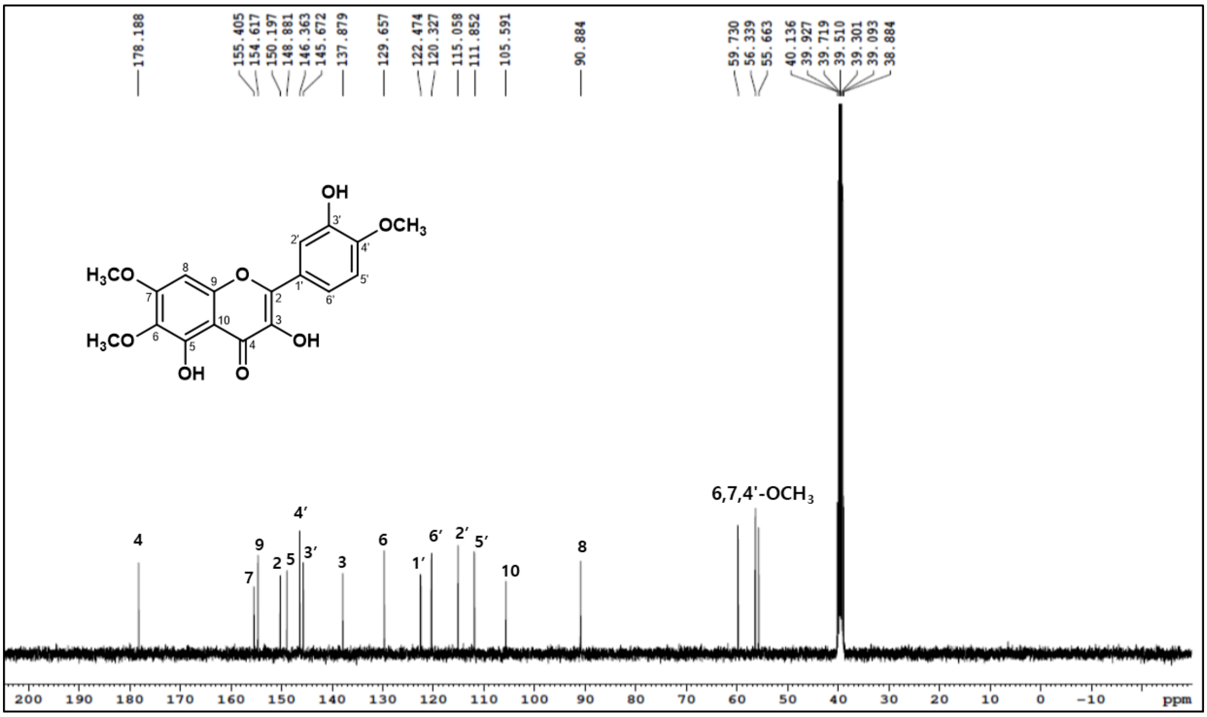

Figure S3. NMR spectrum of eupatin in DMSO-d_6 (400 MHz for ^{1}H NMR, 100 MHz for ^{13}C NMR)
